# Supplementary figures and images for: Comparing in-person, blended and virtual training interventions; a real-world evaluation of HIV capacity building programs in 16 countries in sub-Saharan Africa
Source: PLOS Glob Public Health. 2023 Jul 24;3(7):e0001654. doi: 10.1371/journal.pgph.0001654 (PMC10365303; doi:10.1371/journal.pgph.0001654)

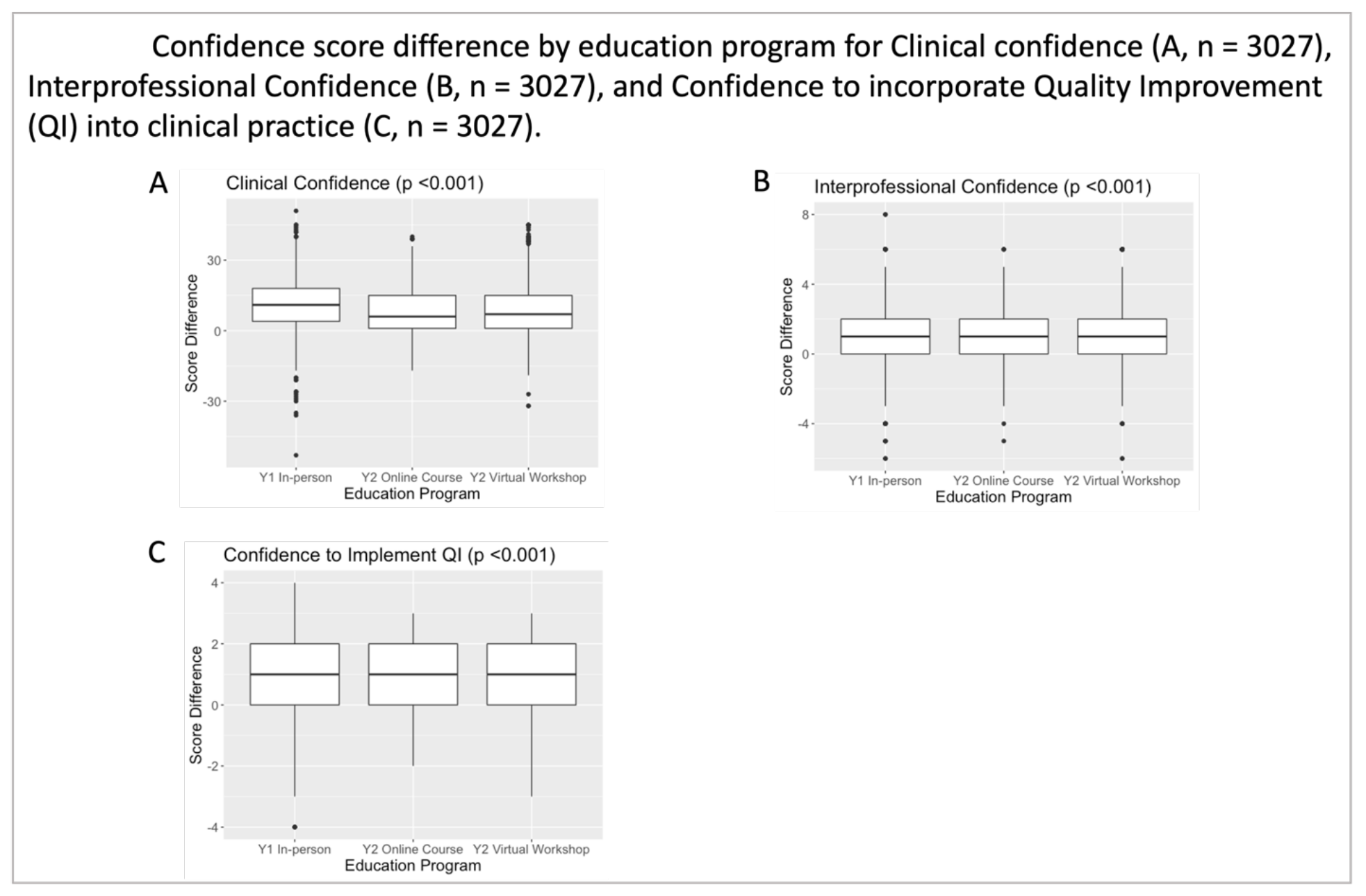

Supplement: S1 Fig — (TIF) [file pgph.0001654.s001.tif]
