# Supplementary material for: Comparing in-person, blended and virtual training interventions; a real-world evaluation of HIV capacity building programs in 16 countries in sub-Saharan Africa
Source: PLOS Glob Public Health. 2023 Jul 24;3(7):e0001654. doi: 10.1371/journal.pgph.0001654 (PMC10365303; doi:10.1371/journal.pgph.0001654)
Supplement: S1 Table — (DOCX) [file pgph.0001654.s002.docx]

**S1 Table.** Assessment questions for each module

| **Category** | **Module** | **Questions** |
| --- | --- | --- |
| **Knowledge** | *New HIV Diagnosis and ART Initiation in a Woman of Childbearing Age* | What do the UNAIDS 95-95-95 goals strive to achieve for people with HIV (PWH)? |
|  |  | Which of the following best describes when to start antiretroviral therapy (ART)? |
|  |  | Which of the following contraception methods may be affected by select antiretroviral therapy (ART) regimens? |
|  | *Management of HIV-TB Co-Infection* | A 26-year-old woman was diagnosed with TB by sputum Gene Xpert testing during a community health campaign three days ago and was sent to your clinic for further evaluation. She was initiated on isoniazid, ethambutol, rifampicin, and pyrazinamide. During your initial evaluation, you recommend an HIV test, which she obtains and turns positive. A point of care CD4 cell count shows 150 cells/mm3. When is the most appropriate time to initiate antiretroviral therapy (ART) for this patient? |
|  |  | Match the following side effects with its most common offending medication. |
|  | *PMTCT & Care for the Pregnant Woman with HIV* | Studies have shown an increased risk of HIV acquisition to women during pregnancy and in the post-partum period. Which of the following reasons could explain the increased risk of HIV acquisition in the post-partum period? |
|  |  | Early infant diagnosis is based on the WHO recommendation that infants born to mothers with HIV be tested for HIV between 4 and 6 weeks of age. Why is early infant diagnosis of HIV important? |
|  | *Care for the Paediatric Patient with HIV* | A 13-month-old girl is brought in by her parent for minimal weight gain. You would like to test her for HIV. Which of the below tests is most appropriate to order in this patient? |
|  |  | When caring for a paediatric patient with HIV, which of the following options for antiretroviral therapy (ART) is best? |
| **Clinical Confidence** | *New HIV Diagnosis and ART Initiation in a Woman of Childbearing Age* | Recognizing the psychosocial implications of a new diagnosis of HIV |
|  |  | Disclosing a positive HIV result to a patient |
|  |  | Providing care for a patient newly diagnosed with HIV as part of a team |
|  |  | Initiating ART for a patient newly diagnosed with HIV, appropriate to my training level. |
|  |  | Providing contraception options for women with HIV |
|  | *Management of HIV-TB Co-Infection* | Starting a patient with HIV-TB co-infection on ART. |
|  |  | Anticipating the toxicities of TB therapy on a patient with HIV-TB co-infection. |
|  |  | Recognizing indications to prescribe TPT (TB preventive therapy). |
|  | *PMTCT & Care for the Pregnant Woman with HIV* | Identifying periods during and after pregnancy that are high risk for HIV acquisition. |
|  |  | Identifying appropriate ART regimens during pregnancy to prevent mother-to-children transmission of HIV. |
|  |  | Identifying strategies for prevention of mother-to-child transmission of HIV in a mother presenting late in pregnancy with a high viral load. |
|  |  | Recognizing the importance of early infant diagnosis. |
|  | *Care for the Paediatric Patient with HIV* | Diagnosing HIV in children less than 18 months of age. |
|  |  | Disclosing HIV status to a child or adolescent. |
|  |  | Recommending ART for children. |
| **IP Confidence** | *Management of HIV-TB Co-Infection* | Caring for a patient with HIV-TB co-infection as part of an interprofessional team. |
|  | *Care for the Paediatric Patient with HIV* | Caring for a child with HIV as part of an interprofessional team. |
| **QI Confidence** | *PMTCT & Care for the Pregnant Woman with HIV* | Using a Fishbone diagram to identify contributors to a systems-based problem. |

*IP*: interprofessional confidence; *QI*: quality improvement. *HIV-TB:* HIV-tuberculosis; *PMTCT:* prevention of mother to child transmission.

1
