# Supplementary material for: Comparing in-person, blended and virtual training interventions; a real-world evaluation of HIV capacity building programs in 16 countries in sub-Saharan Africa
Source: PLOS Glob Public Health. 2023 Jul 24;3(7):e0001654. doi: 10.1371/journal.pgph.0001654 (PMC10365303; doi:10.1371/journal.pgph.0001654)
Supplement: S2 Table — (DOCX) [file pgph.0001654.s003.docx]

**S2 Table**. Maximum score for each assessment category

| **Assessment Category** | **Maximum Score** |
| --- | --- |
| Knowledge Content Domain |  |
| HIV and Women | 3 |
| HIV-TB | 2 |
| PMTCT | 2 |
| Paediatric Care | 2 |
| Total Knowledge Score | 9 |
| Confidence Type |  |
| Clinical | 60 |
| IP | 8 |
| QI | 4 |

IP: Interprofessional Confidence; QI: Quality Improvement Confidence
