# Supplementary material for: Comparing in-person, blended and virtual training interventions; a real-world evaluation of HIV capacity building programs in 16 countries in sub-Saharan Africa
Source: PLOS Glob Public Health. 2023 Jul 24;3(7):e0001654. doi: 10.1371/journal.pgph.0001654 (PMC10365303; doi:10.1371/journal.pgph.0001654)
Supplement: S3 Table — (DOCX) [file pgph.0001654.s004.docx]

## **S3 Table** Total learner baseline mean knowledge score differences for each educational program (Y1 In-person, Y2 Virtual Workshop, and Y2 Online Course) by Total Score, Training Level, and Health Profession compared using Wilcoxon Signed Rank Test

| **Learning Format** |  | **N** | **Mean Pre-Score**  mean (%) | **Mean Post-Score**  mean (%) | **Mean Difference**  Mean (%) | **P-Value** |
| --- | --- | --- | --- | --- | --- | --- |
| **Y1 In-person** | Total Score | 3027 | 5.8 (64.3) | 7.0 (77.9) | 1.2 (13.6) | <0.001 |
|  | Training Level |  |  |  |  |  |
|  | Pre-service learner | 1755 | 5.6 (62.3) | 6.9 (76.4) | 1.3 (14.1) | <0.001 |
|  | Post-graduate new provider | 727 | 5.9 (65.4) | 7.1 (78.8) | 1.2 (13.4) | <0.001 |
|  | Post-graduate provider | 540 | 6.2 (69.3) | 7.4 (81.8) | 1.1 (12.5) | <0.001 |
|  | Health Profession |  |  |  |  |  |
|  | Laboratory | 365 | 5.3 (59.1) | 7.1 (78.5) | 1.7 (19.4) | <0.001 |
|  | Medical | 902 | 6.4 (71.5) | 7.4 (82.2) | 1.0 (10.7) | <0.001 |
|  | Nursing/midwifery | 1145 | 5.5 (61.0) | 6.8 (76).0 | 1.4 (15.0) | <0.001 |
|  | Pharmacy | 312 | 5.9 (65.4) | 7.0 (77.8) | 1.1 (12.3) | <0.001 |
|  | Other | 299 | 5.4 (60.4) | 6.5 (72.0) | 1.0 (11.6) | <0.001 |
| **Y2 Virtual Workshop** | Total Score | 2595 | 6.1 (67.8) | 6.6 (73.7) | 0.5 (6.0) | <0.001 |
|  | Training Level |  |  |  |  |  |
|  | Pre-service learner | 1518 | 6.0 (66.1) | 6.4 (71.2) | 0.5 (5.1) | <0.001 |
|  | Post-graduate new provider | 291 | 6.1 (67.4) | 6.6 (73.9) | 0.6 (6.5) | <0.001 |
|  | Post-graduate provider | 384 | 6.6 (73.3) | 7.3 (80.9) | 0.7 (7.6) | <0.001 |
|  | Health Profession |  |  |  |  |  |
|  | Laboratory | 86 | 5.7 (63.0) | 6.3 (69.7) | 0.6 (6.8) | <0.001 |
|  | Medical | 582 | 6.7 (74.2) | 7.2 (79.9) | 0.5 (5.7) | <0.001 |
|  | Nursing/midwifery | 949 | 5.8 (64.3) | 6.3 (70.1) | 0.5 (5.8) | <0.001 |
|  | Pharmacy | 373 | 6.2 (69.1) | 6.7 (74.1) | 0.5 (5.0) | <0.001 |
|  | Other | 203 | 5.6 (62.7) | 6.2 (68.6) | 0.5 (5.9) | <0.001 |
| **Y2 Online Course** | Total Score | 629 | 5.9 (65.6) | 6.6 (73.2) | 0.7 (7.6) | <0.001 |
|  | Training Level |  |  |  |  |  |
|  | Pre-service learner | 299 | 6.0 (67.2) | 6.7 (74.2) | 0.6 (7.0) | <0.001 |
|  | Post-graduate new provider | 66 | 5.5 (61.0) | 6.2 (68.4) | 0.7 (7.4) | <0.001 |
|  | Post-graduate provider | 158 | 5.8 (64.3) | 6.8 (75.8) | 1.0 (11.6) | <0.001 |
|  | Health Profession |  |  |  |  |  |
|  | Laboratory | 117 | 5.1 (56.2) | 6.3 (69.9) | 1.2 (13.7) | <0.001 |
|  | Medical | 140 | 6.4 (70.8) | 7.2 (80.2) | 0.8 (9.3) | <0.001 |
|  | Nursing/midwifery | 107 | 5.8 (64.3) | 6.2 (68.9) | 0.4 (4.5) | <0.01 |
|  | Pharmacy | 95 | 6.3 (70.5) | 6.9 (77.0) | 0.6 (6.5) | <0.001 |
|  | Other | 64 | 5.8 (64.7) | 6.4 (71.4) | 0.6 (6.7) | <0.001 |

Wilcoxon Signed Rand test used to compare mean pre-scores and mean post-scores in knowledge across the three educational programs. Abbreviations: Y1: year one; Y2: year two; post-graduate new provider: provider participating in training less than 12 months since graduating from health profession training program; Post-graduate provider: provider participating in training at least 12 months or more since graduating from health profession training program; Maximum Score = 9.
