# Supplementary material for: Comparing in-person, blended and virtual training interventions; a real-world evaluation of HIV capacity building programs in 16 countries in sub-Saharan Africa
Source: PLOS Glob Public Health. 2023 Jul 24;3(7):e0001654. doi: 10.1371/journal.pgph.0001654 (PMC10365303; doi:10.1371/journal.pgph.0001654)
Supplement: S4 Table — (DOCX) [file pgph.0001654.s005.docx]

**S4 Table.** Total learner baseline mean confidence score differences for each educational program (Y1 In-person, Y2 Virtual Workshop, and Y2 Online Course) by Total Score and Confidence Type using Wilcoxon Signed Rank Test

| **Learning Format** |  | **N** | **Mean Pre Score**  mean (%) | **Mean Post Score**  mean (%) | **Mean Difference**  mean (%) | **P-Value** |
| --- | --- | --- | --- | --- | --- | --- |
| **Y1 In-Person** | Total Score | 3027 | 51.1 (71.0) | 64.8 (89.9) | 13.6 (18.9) | <0.001 |
|  | Confidence Type |  |  |  |  |  |
|  | Clinical |  | 42.8 (71.3) | 53.9 (89.8) | 11.1 (18.5) | <0.001 |
|  | IP |  | 5.9 (74.3) | 7.3 (91.2) | 1.4 (16.9) | <0.001 |
|  | QI |  | 2.5 (61.4) | 3.6 (90.1) | 1.1 (28.7) | <0.001 |
| **Y2 Virtual Workshop** | Total Score | 2595 | 47.4 (65.9) | 58.3 (80.9) | 10.8 (15.0) | <0.001 |
|  | Confidence Type |  |  |  |  |  |
|  | Clinical |  | 39.6 (66.1) | 48.5 (80.8) | 8.8 (14.7) | <0.001 |
|  | IP |  | 5.5 (68.5) | 6.6 (82.7) | 1.1 (14.3) | <0.001 |
|  | QI |  | 2.3 (58.1) | 3.2 (79.3) | 0.8 (21.2) | <0.001 |
| **Y2 Online Course** | Total Score | 629 | 46.3 (64.4) | 56.6 (78.7) | 10.3 (14.3) | <0.001 |
|  | Confidence Type |  |  |  |  |  |
|  | Clinical |  | 38.7 (64.6) | 47.1 (78.5) | 8.3 (13.9) | <0.001 |
|  | IP |  | 5.4 (66.9) | 6.5 (81.5) | 1.2 (14.6) | <0.001 |
|  | QI |  | 2.2 (56.2) | 3.0 (75.7) | 0.8 (19.5) | <0.001 |

Wilcoxon Signed Rand test used to compare mean pre-scores and mean post-scores in confidence across the three educational programs. Abbreviations: Y1: year one; Y2: year two; IP = confidence in working as part of an inter-professional team; QI = confidence in implementing quality improvement strategies. Maximum Total Score = 72, Maximum Clinical Score = 60, Maximum IP score = 8, Maximum QI score = 4.
