# Supplementary material for: Comparing in-person, blended and virtual training interventions; a real-world evaluation of HIV capacity building programs in 16 countries in sub-Saharan Africa
Source: PLOS Glob Public Health. 2023 Jul 24;3(7):e0001654. doi: 10.1371/journal.pgph.0001654 (PMC10365303; doi:10.1371/journal.pgph.0001654)
Supplement: Attachment — Submitted filename: PGH_033123_RNR_MR_050923.docx [file pgph.0001654.s006.docx]

Center for Global Health Delivery, Diplomacy & Economics
Institute for Global Health Sciences | UCSF
550 16th Street, 3rd Floor
San Francisco, California 94158

May 9, 2023

Dear Editors,

*Re: PGPH-D-23-00199, Comparing in-person, blended and virtual training interventions; a real-world evaluation of HIV capacity building programs in 16 countries in sub-Saharan Africa*

Thank you for considering our manuscript for publication in PLOS Global Public Health. We appreciate the thoughtful comments and feedback provided by the reviewers and the editorial team.

We have carefully considered the comments and have made revisions to address the points raised during the review process. We have included a wider range of recent literature on the topic and have provided a detailed consideration of how participant differences affect the results, in addition to differences in training.

Furthermore, we have worked to clarify the interpretation of pre/post scores and have taken caution in generalizing from our study to online training in general.

Finally, we have sought to address all of the comments from your reviewers. These responses are outlined below. We hope that these revisions have adequately addressed the concerns raised, and we appreciate the opportunity to revise and resubmit our manuscript. Thank you for your time and consideration.

Sincerely,

Dr. Mike Reid, on behalf of the authors

Response to Reviewer #1:

***Reviewer: The background discusses the need to switch as well as the limited evaluation to date. There is now a wealth of literature, although many of these may have been after submission. This should be updated.***

Response #1.1: We appreciate the reviewer's recognition of our study's strengths, and agree that updating our literature review was necessary to reflect new studies conducted since submission. We have revised the literature review section to include more recent studies. These include new citations, such as the following:

Kagawa MN, Chipamaunga S, Prozesky D, Kafumukache E, Gwini R, Kandawasvika G, et al. Assessment of Preparedness for Remote Teaching and Learning to Transform Health Professions Education in Sub-Saharan Africa in Response to the COVID-19 Pandemic: Protocol for a Mixed Methods Study With a Case Study Approach. JMIR Res Protoc. 2021;10(7):e28905.

Marsh RH, Plyler C, Miller M, Klar R, Adeiza M, Wachekwa I, et al. Facing COVID-19 in Liberia: Adaptations of the Resilient and Responsive Health Systems Initiative. Ann Glob Health. 2021;87(1):101.

Ogbenna A, Drane D, Crowe AN, Oyedele O, Hauser J, Soyannwo O, et al. Building the Nigerian Palliative Care Workforce: An Interdisciplinary Distance Learning Training Program. Ann Glob Health. 2022;88(1):96.

Downie A, Mashanya T, Chipwaza B, Griffiths F, Harris B, Kalolo A, et al. Remote Consulting in Primary Health Care in Low- and Middle-Income Countries: Feasibility Study of an Online Training Program to Support Care Delivery During the COVID-19 Pandemic. JMIR Form Res. 2022;6(6):e32964.

Khoza-Shangase K, Moroe N, Neille J. Speech-Language Pathology and Audiology in South Africa: Clinical Training and Service in the Era of COVID-19. Int J Telerehabil. 2021;13(1):e6376.

***Reviewer: The details in analysis at the bottom of the tables belongs in methods. The decision of which option was made by the institution and a discussion of differences in those settings as well as the providers they train would have been important beyond the individual level differences.***

Response #1.2: Thank you for the suggestion. We have also provided a more in-depth description of how assessment of ‘confidence’ was determined in the methods, rather than as a footnote to Table 3. In the methods section we have added the following additional details:

*We evaluated confidence among participants by assessing the mean difference in pre-test and post-test confidence scores in three arenas: clinical confidence, confidence in working as part of an inter-professional team (IP Confidence), and confidence in implementing quality improvement strategies (QI Confidence). Across these three confidence domains, participants were invited to complete a series of questions asked on a Likert scale of 1 (“I feel uncomfortable with this topic/need supervision from my supervisor”) to 4 (“I feel very comfortable with this topic/without supervision as though in independent practice”). Fifteen questions assessed clinical confidence, two questions assessed IP confidence and one question addressed confidence to leverage QI tools in clinical practice. These confidence scores were summed for each participant, and the mean differences in confidence scores were compared across the three educational programs, stratified by health profession and training level, using Analysis of Variance (ANOVA) and Tukey’s Honest Significance tests as outlined below.*

In addition, we have explained that different partner institutions chose to target their training programs to different learners per their preference based on convenience, resources and type of training institution. A more detailed description of how different institutions targeted STRIPE training to different learners is outlined in our earlier analysis:

Kiguli-Malwadde E, Forster M, Martin S, Chilemba E, Couper I, Motlhatlhedi K, et al. Evaluating the impact of a multicountry interprofessional training programme to improve HIV knowledge and clinical confidence among healthcare workers in sub-Saharan Africa: a cohort study. BMJ Open. 2022;12(7):e060079.

In the revised draft, we have summarized the process for learner recruitment and participation:

*“As outlined in our previous work, each STRIPE collaborating institutions recruited learners from different cadres (e.g. nursing, medicine, pharmacy, etc.), stages of professional development (pre-service, early graduate, etc.) and clinical settings (clinic, hospital, classroom, etc.) according to local training priorities. Moreover, collaborating institutions employed different, non-standardized recruitment strategies to optimize the participation of learners, with some institutions providing airtime or access to Internet to learners who participated in VW or OC courses.”*

***Reviewer: There were multiple comparison between demographics, but no discussion of multiple tests.***

Response #1.3: We appreciate this feedback. In the submitted draft we had highlighted in the ‘statistical analysis subsection of the methods section what statistical tools we used. However, in the revised version we have clarified which of these tools for each of the different comparisons. This includes the following statement:

*“We compared learner demographics using Pearson chi-squared tests. Wilcoxon signed rank test was used to identify baseline pairwise differences between the pre-test and post-test means for each educational program overall, as well as by training level and health profession. We used Analysis of Variance (ANOVA) and Tukey’s Honest Significance Test (HSD) to compare mean differences in knowledge and confidence between the three educational programs. Analyses were carried out using RStudio (2022.07.1+554). Additionally, p<0.05 was considered statistically significant.”*

***Reviewer: When describing the results, % is needed as well as numbers, and ideally some description if available of the differences between people who responded and who did not.***

Response #1.4: Thank you for your feedback. We have added percentages and changes in absolute scores where appropriate in the narrative. In the original submission, we had summarized the numbers for each of the groups for whom we had all we complete demographic data. In response to this reviewer feedback we went back and reviewed the outcomes data both those individuals whose demographic was not available in the three groups (IP, VW and OC). As noted in the manuscript, complete demographic data was available for 3022, 2193 and 527 learners in the In-person, VW and OC respectively, while pre- and post-course evaluative data was available for 3027, 2595 and 629 participants in each of these groups. In a sensitivity analysis, which we have included in the appendix, we found no differences in mean scores for knowledge or confidence between those for whom we had demographic data and those we did not, regardless of whether they were in the IP, VW or OC group. This additional analysis is included here:

*“There was a small proportion of participants for whom we had complete demographic data but not evaluative data. We undertook a sensitivity analysis to determine if those participants for whom we did not have complete demographic records responded differently to the pre- and post-surveys from those for whom we did not have complete demographic data. In this additional analysis, we found no differences in mean scores for knowledge or confidence between those for whom we had demographic data and those we did not, regardless of whether they were in the IP, VW or OC group.”*

***Reviewer: The baseline scores on knowledge and confidence across the 3 groups would also help understand additional differences****.*

Response #1.5: Thank you for the suggestion. In the revised submission we have included baseline scores on knowledge and confidence for the three groups. Since this is not the primary focus of the paper, this data is included in the Supplemental file, and referenced accordingly in the main manuscript.

***Reviewer: I assume that the differences are absolute but this should be defined.***

Response #1.6: We have clarified in the results section that the differences reported are absolute differences in the footnotes for both Table 2 and 3.

***Reviewer: Consideration of a MVA would have helped understand if the differences were due to the mode versus the attendees.***

Response #1.7: Thank you for the suggestion. We deliberated greatly as to whether a multivariate analysis would confer substantial additional insights. Ultimately, we felt that univariate and bivariate analyses were sufficient to understand the relationships between assessment scores and demographic variables. Moreover, as pointed out in the analysis the ANOVA test allowed us to accurately assess differences in the groups, including when controlling for relevant variables.

***Reviewer: For the confidence, was there any differences in attendance at the relative sessions (or were those 4 the ones related to the confidence).***

Response #1.8: Thank you for the comment. The four modules were compulsory for learners, regardless of what type of training they enrolled in. Moreover, we only included those individuals for whom complete data was available on the four modules. As such we cannot make any comments on the difference in attendance on different platforms.

***Reviewer: The first sentence that were “similarly effective” does not reflect the main findings, although it is clear that the differences may not have been proportionally difference (relative versus absolute change). The first sentence of paragraph 2 seems incomplete.***

Response #1.9: Thank you for your feedback. We have revised the first sentence of the discussion to more accurately reflect the findings. The revised manuscript now reads as follows:

*“In this multi-country capacity-building intervention, which included over 13,000 learners from diverse cadres and clinical settings, in-person and different online educational strategies all led to significant improvements in knowledge and clinical confidence.”*

We have also revised the first sentence of the second paragraph for clarity. The sentence now reads as follows:

*Firstly, it was notable that the magnitude of gains in technical knowledge and clinical confidence were smaller among learners who participated in the synchronous Virtual Workshop and blended Online Course compared to those in the in-person training.*

***Reviewer: The virtual workshop has breakouts, while the online classroom and in-person did not. So comparison of the 2 virtual approaches needs to address differences in difference forms of pedagogy. References to other work for transition to online training during COVID as well as the established literature on online training is needed.***

Response #1.10: Thank you for the suggestion. We have added a discussion of the different forms of pedagogy used in the virtual workshop versus the online classroom and in-person training, and how this may have impacted the results. We have also added references to other work for transition to online training during COVID and the established literature on online training. However, as a point of clarification, both the virtual and online formats included breakouts.

***Reviewer: I think we want to take great care in calling training effective when we are not truly measuring the goal of training - change in provider performance to improve health outcomes. I think we must be very careful not to give the impression that virtual learning is the panacea we've all been waiting for. This paper does not say that but I believe it could be a bit stronger in saying what research is needed and what we cannot say with knowledge and confidence assessments. You'll see below specific places you could consider strengthening that language.***

Response #2.1: Thank you for the feedback. We agree that it is important to carefully describe the limitations of our study, and to acknowledge that changes in knowledge and confidence may not directly translate to changes in provider performance or health outcomes. We have revised the discussion section to more clearly state the limitations of our study, and to provide suggestions for future research. These include the following edits:

*“The study has several limitations. Firstly, data is from an HIV-specific training program, and evaluative data limited to learners who completed the registration survey and assessment questions. Further research is warranted to determine if lessons learned from the analysis are applicable to other disease-specific capacity-building interventions. The three different approaches employed different pedagogical techniques (for example both in-person and virtual workshops included small group breakouts) that might have impacted learner outcomes as much as the medium of delivery (online vs. in-person). More health professions education research to understand how best to employ digital tools in Africa is a priority that has been highlighted by numerous stakeholders. ^1-4^ Additionally, our assessment was limited to knowledge and confidence, as data regarding clinical outcomes was not available. Since different health professional training institutions participated in the program in Y1 and Y2 (in part due to digital literacy and internet infrastructure), we were unable to compare learner outcomes between Y1 and Y2 within individual institutions or country.”*

***Reviewer: Interesting but not surprising finding that online = more equity in some respects. However where bandwidth is not good, virtual learning is inaccessible so a different type of inequity.***

Response #2.2 This is a valid point and one that we have highlighted in the limitation section. This revision is outlined below:

In Y2, we only included information from learners that were able to participate in the digital modalities highlighted here; the study does not address barriers faced by learners unable to access online training programs in SSA. These limitations highlight substantial digital inequities in health professions training institutions between and within countries in SSA and underscore the ongoing need to support the development of local technological infrastructure for online education across SSA. The limitations section highlights this:

*“Finally, institutions in Y2 were different from Y1 since participation was limited by digital literacy and Internet infrastructure. In Y2, we only included information from learners that were able to participate in the digital modalities highlighted here; the study does not address barriers faced by learners unable to access online training programs in SSA. These limitations highlight substantial digital inequities in health professions training institutions between and within countries in SSA,^5,6^ and underscore the ongoing need to support the development of local technological infrastructure for online education across SSA.”*

***Reviewer: I would list the countries after "participated.” hitherto means before - I believe you might use hereafter?***

Response #2.3: Thank you for the suggestions. We have revised the manuscript to reflect these changes, including listing the countries on page 8, using "hereafter" instead of "hitherto," and correcting the sentence on page 9.

***Reviewer: "To compare the three learning approaches, we evaluated data from four training four modules that utilized" Did you mean to use four twice?***

Response #2.4: This was a typo and we have corrected it.

***Reviewer: Is the change in confidence less in post grads likely b/c post grad start more confident? If so, would be good to mention and discuss.***

Response #2.5: We know from previously published work that post-graduate learners have greater pre-participation clinical confidence. We focus on how the the approach to learning influences the change in confidence in this analysis. We are cautious about making too many inferences about the pre-participation conference was different between the three approaches because there were many extenuating circumstances in Y2 (e.g. COVID-19 pandemic) that may have influenced learner confidence.

***Reviewer: Greater improvement in knowledge gains in-person compared to VW/OC seems to show the value of in person. OR is it really the difference in how many hours for in person vs online meaning engagement with the learning? OR changes in materials? You describe the tests as being the same but a bit of a description of # of hours of each recognizing that you would have to estimate as asynchronous learning varies. For in person I believe you said 2 days so is this 16 hours? Similarly, how were the materials changed/adapted for the VW/OC. If they were shortened, this could explain some of the differences you saw.***

Response #2.6: Thank you for your feedback. This is a great insight. While in the in-person training is longer this is because more modules are included. However, we only included data for 4 standard modules for which learning objectives and assessment tools were the same. Moreover, the way the modules were designed, we would have anticipated that learners spent roughly the same amount of time on each module regardless of how the module was taught. Obviously, its possible that when taught in person, learners spent longer on these modules, but we have no reason to presume that this was the case. We have sought to clarify this in the revised draft in the ‘study and design’ sub-section of the methods section.

***Reviewer: Interesting that the difference for clinical confidence is huge. Might be worth discussing? Were the training materials more geared to the clinical components? Probably the biggest area that I'd like to see fleshed out a bit more concerns changes in knowledge and confidence being equated with efficacy. The literature is replete with examples showing improvements in knowledge and even skills after training as compared to before but then NO change in provider performance or outcomes occurs.***

Response # 2.7. Thank you for this comment. We have added a caveat to our discussion section highlighting that we are hesitant to draw any conclusions as to the impact of the training on clinical outcomes based on our results. Moreover, the focus of this analysis was primarily to compare the different modalities for learning rather than make inferences on the impact of changes in clinical confidence on clinical outcomes, which we intend to evaluate in a separate, distinct analysis. We have included the following revision, in response to this comment:

*“Firstly, it was notable that the magnitude of gains in technical knowledge and clinical confidence were smaller among learners who participated in the synchronous Virtual Workshop and blended Online Course compared to those in the in-person training. Across all domains of confidence assessed, mean improvements in subjective confidence were greatest among learners who participated in the in-person program, regardless of cadre and career stage. We are cautious drawing conclusions about the implications of these findings on clinical outcomes since this was not assessed in this study. Nonetheless, these findings validate other research demonstrating the importance of online tools as satisfactory for acquiring knowledge, but inferior modalities for acquisition of clinical skills or technical confidence.^7,8^ These findings underscore the importance of in-person learning for inter-professional training. They also highlight the potential limitations of on-line training, which may be considerable, if deficiencies in knowledge and confidence lead to adverse clinical outcomes.”*

***Reviewer: "in-person and different online educational strategies were demonstrated to be similarly effective means of training learners in inter-professional HIV care across SSA." Effective by what metric? So the two you measured yes but I think we have to state early and often that this does not automatically equate to efficacy for the real goal which is improving care for patients with HIV.***

Response #2.8. Thanks for this comment. We have revised this section of the discussion accordingly, and in response to a similar comment from Reviewer #1. The revised paragraph reads as follows:

*“In this multi-country capacity-building intervention, which included over 13,000 learners from diverse cadres and clinical settings, in-person and different online educational strategies all led to significant improvements in knowledge and clinical confidence.”*

***Reviewer: "Moreover, the transition from in-person to online and remote training that occurred because of the COVID-19 pandemic, did not have a major deleterious impact on learner outcomes, as demonstrated by the gains in both knowledge and confidence across all three educational programs and for all types of learners." But the gains were not as good for remote on knowledge and we don't know about the learner outcome of change in performance.***

Response #2.9. This is a valid point. In the revised submission, we have added several sentences to the second paragraph of the discussion as outlined here:

*“We are cautious drawing conclusions about the implications of these findings on clinical outcomes since this was not assessed in this study. Nonetheless, these findings validate other research demonstrating the importance of online tools as satisfactory for acquiring knowledge, but inferior modalities for acquisition of clinical skills or technical confidence. These findings underscore the importance of in-person learning for inter-professional training. They also highlight the potential limitations of on-line training, which may be considerable, if deficiencies in knowledge and confidence lead to adverse clinical outcomes.”*

***Reviewer: "For example, online learning tools are likely to be more affordable in many parts of SSA" Yes, but I think we need to be careful stating this until we have knowledge that the effect on patient care and outcomes is similar. Something may be affordable but it it's not effective it will never be cost-effective.***

Response #2.10. This is an excellent point. We have revised the language in this paragraph to highlight the importance of more rigorous analysis, both to assess the impact of new training interventions on clinical outcomes before, and in addition to, analyses related to cost-effectiveness.

***Reviewer: "These benefits notwithstanding,(17) more research is warranted to ensure that online capacity building interventions are used effectively, and in combination with traditional inperson strategies, to foster clinical confidence and inter-professional collaboration." I agree 100% but please add something about competence and/or change in provider performance.***

Response #2.11. In response to this excellent insight we have revised the manuscript to highlight the importance of more research evaluating the impact of these training interventions on provider performance. These additions are highlighted here:

*“These benefits notwithstanding, more research is warranted to ensure that online capacity-building interventions are used effectively, and in combination with traditional in-person strategies, to foster clinical confidence and inter-professional collaboration, and determine if they lead to improvements in provider performance and superior clinical outcomes.”*

***Reviewer: "Although many health professions training institutions in SSA lack access and capacity to use digital technologies to deliver HIV training(20) our results affirm the critical role that online training interventions can play in advancing professional development opportunities especially for those healthcare professions historically underserved by capacity building interventions.(21)" Yes, but we need to ensure that the end goal - improving patient care through change in provider behaviors - is achieved. So many donors and even ministries now are saying that training can all be done virtually and we know that this is not an accurate statement. I would never train someone to conduct a birth, manage a hemorrhage, use a vacuum for an assisted delivery exclusively online. However how far can we go to decrease the face to face time through blended learning? Blended approaches are indeed needed and can be beneficial. What is is the right balance of in person vs virtual? While HIV care is likely much less skill driven as it is adherence to a protocol or treatment algorithm, something made in person learning more effective at knowledge acquisition.***

Response #2.11. Again, we greatly appreciate the perspective of the reviewer and agree with their conclusion. In the revised discussion we have highlighted the value of blended learning approaches to enhance knowledge, but also inter-professional communication and collaboration. However, we note that existing digital tools have substantial limitations, especially with respect to certain forms of procedural instruction, etc.
